# Characterization of the complete mitochondrial genome of the Reeves’ muntjac *Muntiacus reevesi* (Artiodactyla, Ruminantia, Cervidae) and its phylogeny

**DOI:** 10.1080/23802359.2021.2015267

**Published:** 2021-12-28

**Authors:** Yi Huang, Qin Gao, Liping Mei, Linghan Lei, Jingtian Yang, Jinyao Hu

**Affiliations:** a Engineering Research Center for Forest and Grassland Disaster Prevention and Reduction, Mianyang Normal University, Mianyang, P. R. China; b Juyuan High School of Dujiangyan, Dujiangyan, P. R. China

**Keywords:** *Muntiacus reevesi*, Cervidae, mitogenome, genome organization, phylogenetic analyses

## Abstract

We present the complete mitogenome of *Muntiacus reevesi*. We found that the mitogenome of this circle is 16,535 bp in size and includs 13 protein-coding genes, 22 transfer RNA genes, two ribosomal RNA genes, and one noncoding control region (D-loop) that are conserved in most Cervidae mitogenomes. The total base composition of the Muntiacus reevesi mitogenome is 33.18% A, 28.99 % T, 24.43% C, and 13.40% G, which is typical for mammalian mitogenomes. Phylogenetic analyses. Phylogenetic analyses showed that *M. reevesi* clustered with *M. vuquangensis* and *M. putaoensis* as a branch and that they are closely genetically related.

The Reeves’ muntjac (*Muntiacus reevesi*) is a small deer species, belonging to *Muntiacus*, Muntiacinae, Cervidae. This species is endemic to China including Taiwan Island. In mainland China, this species ranges from Guangdong and Guangxi up to Gansu and Shaanxi, covering the vast subtropical region of the Zhujiang (Pearl) and Yangtze River catchment basins (Timmins and Chan 2016). It is highly adaptable and can be found in temperate forests with occasional snowfall as well as in dense forests in the warm subtropical zone (Timmins and Chan 2016). The complete mitogenome has proven to be a highly effective resource for studying the genus *Muntiacus* species evolution and population genetics (Li et al. 2017; Kuang et al. 2019). However, molecular studies on the Reeves’ muntjac were limited and the genetic relationship between Reeves’ muntjac and related species is still vague. We, therefore, report here the complete mitogenome of *M. reevesi* and clarify its relationships with related species of the genus *Muntiacus*.

Specimens of *M. reevesi* were collected in August 2020 from Tangjiahe Natural Reserve, Qingchuan County, Sichuan province, China (104°45′34.23″E, 32°35′14.72″N), and immediately preserved in 95% ethanol at −75 °C until use. The specimen was deposited at the Ecological Security and Protection Key Laboratory of Sichuan Province, Mianyang Normal University (http://zdsys.mnu.cn/; Yi Huang; hyhy1232021@163.com) under the voucher number HY2020082203. Total DNA was extracted following the method of Sambrook and Russell (2001). We employed polymerase chain reaction (PCR) and Long-and-Accurate PCR methods to amplify the complete mitogenomic region of *M. reevesi* with the PCR primers designed by Hassanin et al. (2009) and ourselves. The reaction protocol, amplification system, and sequencing were carried out using Hassanin et al’s (2009) and Jiang et al.’s (2013) methods. The sequence was submitted to GenBank with the accession number MZ895085.

Specimens of *M. reevesi* were collected from Tangjiahe Natural Reserve, Qingchuan County, Sichuan province, China in August 2020 (104°45′34.23″E, 32°35′14.72″N), and immediately preserved in 95% ethanol at −75 °C until use. The specimen was deposited at the Ecological Security and Protection Key Laboratory of Sichuan Province, Mianyang Normal University (http://zdsys.mnu.cn/; Yi Huang; hyhy1232021@163.com) under the voucher number HY2020082203. Total DNA was extracted following the method of Sambrook and Russell (2001). We employed polymerase chain reaction (PCR) and Long-and-Accurate PCR methods to amplify the complete mitogenomic region of *M. reevesi* with the PCR primers designed by Hassanin et al. (2009) and ourselves. The reaction protocol, amplification system, and sequencing were carried out by Hassanin et al.’s (2009) and Jiang et al.’s (2013) methods. The sequence was submitted to GenBank with the accession number MZ895085.

The mitogenome of *M. reevesi* has a total length of 16,535 bp, and the base composition is 33.18% A, 28.99% T, 24.43% C, and 13.40% G, respectively. The whole mitogenome consists of 13 protein-coding genes, 22 tRNA genes, 2 rRNA genes, and 1 control region. The mitogenome of *M. reevesi* shows the typical gene content observed in mammalian mitogenomes (Hassanin et al. 2012; Hong et al. 2017; Liu and Zhang 2018). Eight tRNAs are encoded on the light strand (*tRNA-Asn*, *tRNA-Glu*, *tRNA-Tyr*, *tRNA-Gln*, *tRNA-Pro*, *tRNA-Cys*, *tRNA-Ser*, and *tRNA-Ala*). Only one PCG is encoded on the light strand (ND6), whereas the other genes are located on the heavy strand. ATG was used as the starting codon for most protein-coding genes, except for *ATA* in *ND2* and *ND3* and *GTG* in *DN4L*. *Cytb* and *DN2* genes terminated with *AGA* and *TAG*, *ND3*, *ND4*, and *COIII* genes terminated with an incomplete stop codon (T−), and other protein-coding genes terminated with TAA.

To evaluate the phylogenetic relationships between *M. reevesi* and the other related species, phylogenetic trees were rebuilt using BI and ML methods based on the nucleotide dataset (Alam et al. 2010; Yu et al. 2012). The most suitable TIM2 + I + G model was selected in jModelTest 0.1 (Darriba et al. 2012), and the same phylogenetic tree was obtained with high node support, containing the reported 30 mitogenome sequences of Cervinae (Figure 1). According to the phylogenetic tree, *M. reevesi* clustered with *Muntiacus vuquangensis* and *Muntiacus putaoensis* into a branch and they have a close genetic relationship. Monophyletism of *Mununtiacus*, *Elaphodus*, *Aix*, and *Dama* is well supported and has been reported in other recent studies (Li et al. 2017; Singh et al. 2019; Zhang et al. 2019). In this study, we present the complete mitogenome sequence of *M. reevesi*, which would contribute to further phylogenetic analysis of this species. And more mitogenomic data of undetermined taxa and further analysis are required to reveal phylogeny and evolution of Cervidae.

**Figure 1.**
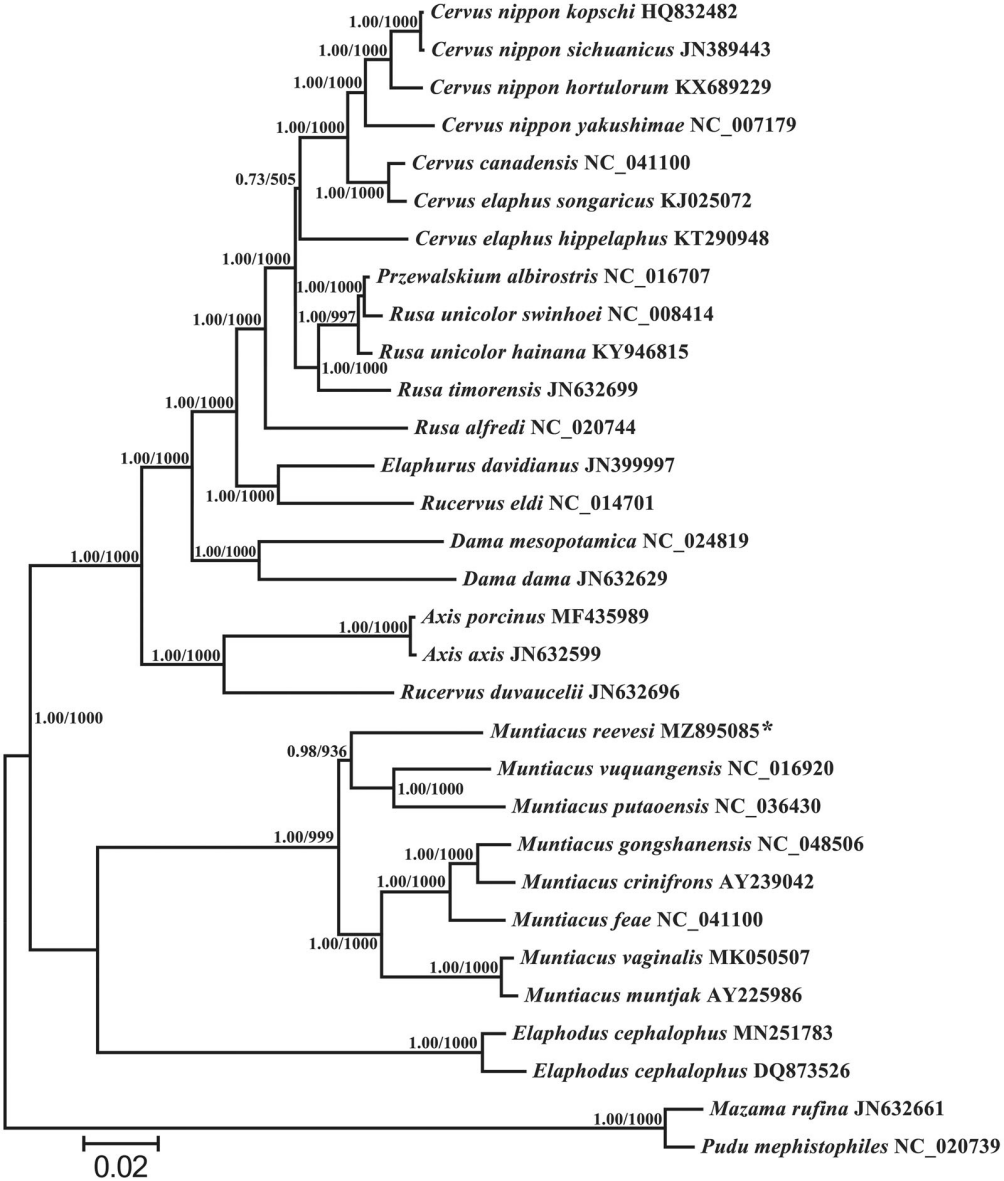
Based on the nucleotide data set of 13 mitochondrial protein-coding genes, a phylogenetic tree of 30 reported mitogenome sequences between Cervinae and two Odocoileinae outgroups (*Mazama rufina* and *Pudu mephistopheles*) were established. The branch length and topology were derived from BI analysis. Numbers above branches specify posterior probabilities from Bayesian inference (BI) and bootstrap percentages from maximum likelihood (ML, 1000 replications) analyses. Tree topologies produced by Bayesian inferences (BI) and maximum likelihood (ML) analyses were equivalent. bootstrap support values for ML analyses and Bayesian posterior probability are shown orderly on the nodes. The asterisks indicate the new sequences generated in this study.

## Data Availability

The mitogenome sequence data that support the findings of this study are openly available in GenBank of the NCBI at (https://www.ncbi.nlm.nih.gov/) under accession no. MZ895085. The associated BioProject, SRA, and Bio-Sample numbers are PRJNA764861, SRR15992505, and SAMN21531723, respectively.
